# Differences between Elite and Semi-Elite Australian Football Conceptualised through the Lens of Ecological Dynamics

**DOI:** 10.3390/sports7070159

**Published:** 2019-06-28

**Authors:** Carl T. Woods, James Jarvis, Ian McKeown

**Affiliations:** Football Department, Port Adelaide Football Club, South Australia, Adelaide 5014, Australia

**Keywords:** skill acquisition, ecological dynamics, performance analysis, non-linear pedagogy, interdisciplinary research

## Abstract

This study explored the differences in match play between elite and semi-elite Australian football (AF) conceptualised through the lens of ecological dynamics. We sampled naturalistic constraints from match play across two AF competitions (elite and semi-elite) and heuristically classified them into *task*, *environmental* and *individual* classes. Data was extracted from 22 Australian Football League (AFL) games, and 18 semi-elite AF games, with a total of six constraints being sampled from each game. Match play within the AFL generated a greater percent of total disposals in general play within a processing time of 0–1s (*d* = 1.24 (0.64–1.80)), a greater opposition density surrounding the ball carrier (*d* = 0.82 (0.26–1.37)), and more disposals being performed while running (dynamic; *d* = 0.89 (0.33–1.45)). This data highlights differences with regards to the informational sources available to players across both competition standards to inform their movement choices. Specifically, a greater proportion of disposals within the AFL appear to be shaped by pronounced temporal and spatial constraints relative to a semi-elite competition. Coaches are encouraged to consider these results when developing representative training activities for both AFL and prospective AFL players.

## 1. Introduction

Ecological dynamics is a contemporary theory of skill acquisition, positing that skilled behaviour is the emergence of co-adaptive, functional behaviours that satisfy a unique set of interacting constraints impacting upon a system [1]. Accordingly, these constraints shape coordinative patterns within human movement systems as they act as “boundaries” within which movement solutions emerge [2]. One of the most recognised constraint-based models of the human movement sciences is that proposed by Newell [3] who categorised constraints into three distinct classes: Organismic (or performer/individual), environmental or specific to the task. Organismic constraints are those relating to structural (e.g., standing height or buoyancy), or functional (e.g., speed, endurance, resilience or motivation) components residing within an individual [4]. Environmental constraints are those viewed externally to the human movement system and include things such as the temperature, altitude or surface in which an activity is performed on [5]. Task constraints are those specific to the action or activity to be performed, with examples being a game’s outcome, rules or equipment used [6].

Given the recent expansion of technology and statistical techniques applied to the sports sciences, sports performance analysts and skill acquisition specialists have sought to identify key variables believed to enhance training and game design through the use of notational analysis [7]. However, a common criticism of this work has been its lack of theoretical rationale [8], and innate focus on the quantification of a movement’s outcome, as opposed to sampling the constraints that shape the quantified action [9]. When conceptualized through ecological dyanmics, sampling constraints that shape human behaviour would likely provide evidence to improve representative learning design, guide the incorporation of functional variability in training, and assist with an athlete’s attunement to relevant affordances (opportunities for action) during competition [1,10].

Constraints-based analysis of training specificity has recently been applied to Australian football (AF) [11]. By sampling individual (movement characteristics at point of ball disposal), environmental (opposition density around ball carrier) and task (possession time) constraints in both training and game contexts, Corbett et al. [11] generated a training prescriptive system that determined the similarity of training drills, as well as their specificity to match play. Such an approach is likely to assist a coach with the design or manipulation of a training activity to increase its representativeness relative to the constraint’s (or informational sources) players experience during match play. Based on this work, we hypothesise that sampling constraints which are believed to shape behaviours within team sport may allow insights into competition differences. Specifically, the comparison of key constraint characteristics across differing competition standards (e.g., elite and semi-elite) may offer insight into the performance environments athletes are afforded to shape their movement solutions. This information may subsequently be used by coaches to contextualise observed behaviours in both competition standards relative to the constraints that shape the emergent behaviours. The outcomes of such findings would likely extend to the informed design of training activities that offer closer representations to what semi-elite players may experience in elite senior competitions; thus, assisting with talent development.

Guided by Newell’s [3] constraints-led approach, the aim of this study was to sample and compare selected constraints impacting ball disposal in elite (Australian Football League; AFL) and semi-elite AF competitions. We hypothesised that the constraints shaping disposals within the AFL would meaningfully differ to those sampled from the semi-elite competition given the postulated greater physical, technical and perceptual capacities of AFL players. An associated outcome of this work was to encourage sports performance analysts to embrace interdisciplinary collaboration, and align practices with theories common to skill acquisition.

## 2. Methods

A convenience sample of 45 players contracted to the same AFL club participated in this study. All players competed within at least one AFL (elite) or state-based (semi-elite) match, with 19 competing within both the AFL and state-based competitions given uncontrollable team selection strategies implemented by the clubs coaching staff. Informed consent was provided by the relevant football club, with ethical declaration granted by the relevant Human Research Ethics Committee.

### 2.1. Procedures

This study followed a longitudinal, cross-sectional observation design, and was conducted over the course of one regular AFL season. Data was sampled from 22 AFL and 18 state-based matches (n = 40 games) via the use of video footage from three, two dimensional, cameras (Sony FS7, Panasonic AG-HPX372EN, Osaka, Japan). Two of the cameras were in an aerial position behind the goals, while the third was placed in an aerial position perpendicular to the playing field (broadcast view). To sample match play constraints associated with each possession and subsequent disposal pair of events, notational analysis software was used (Sportscode version 11.2.18, Sportstec Inc. Warriewood NSW, Australia). Six constraints were then sampled from each game, categorised into task (n = 3), environmental (n = 2) and individual (n = 1) classes. The three task constraints we sampled oriented possession time and disposal location. Possession time was calculated as the time between the player first obtaining ball possession to the point in which they disposed of it. As described in Table 1, we then categorised this into two components—possession time in general play and possession time from a stoppage in play. Possession times were then split into four temporal epochs, measured via Sportscode’s timer feature. Disposal location was spread into four field zones common to AF—defensive 50, defensive mid, attacking mid and forward 50 (Table 1). This was defined as the zone in which the player disposed of the ball. Secondly, two environmental constraints were sampled, orienting opposition density surrounding the disposal target and the ball carrier (Table 1). Both environmental constraints were defined by the number of opposition players within a 3m radius of either the target or ball carrier. Lastly, the individual constraint was defined via the movement dynamics of the player at the point of ball disposal; dichotomised into stationary and dynamic categories (Table 1). These constraints, their descriptions and sub-categories were heuristically chosen based on skill acquisition specialist consultation and recommendations from prior work [11]. 

The video footage was stacked from each perspective and exported into Sportscode for analysis. Each match was coded by the same analyst and their intra-rater reliability was assessed by randomly coding 10% of the sample on two separate occasions (spaced by at least seven days). The Kappa statistic (k) was used to measure the intra-rater reliability [12]. The agreement levels of the intra-rater reliability for each constraint ranged from “moderate” (k = 0.41) to “substantial” (k = 0.80).

### 2.2. Statistical Analysis

All statistical analyses were done using R (version 3.2.5, Vienna, Austria) [13]. All data were transformed to represent a percent of total disposals performed within each constraint. The mean and standard deviation was calculated for each constraint (explanatory variables) and sorted relative to competition (two levels: Elite, semi-elite). The effect size and subsequent 90% confidence interval were then calculated using Cohen’s d statistic in the “MBESS” package [14]. The effect size interpretations were in accordance with recommendations provided elsewhere [15].

## 3. Results

**Figure 1:**
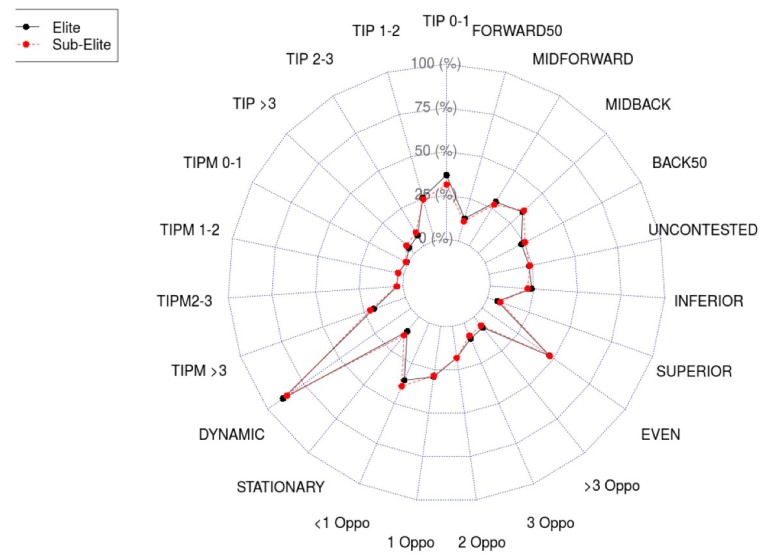
Radar plot showing the percent of total disposals performed within each constraint category. Note: “Oppo” denotes opposition, “TIP” denotes time in possession, “TIPM” denotes time in possession from mark

The descriptive statistics and effect sizes for each constraint are presented in Table 2, and have been visualised in Figure 1. The largest between group effects can be seen within the general play possession time constraint categories, with the percent total disposals performed within the 0–1s, 2–3s and >3 s epochs each possessing a large effect (Table 1). Further, AFL game play generated a greater percent of total disposals with a density of three or more oppositions players surrounding the ball carrier (Table 2), while fewer disposals were performed within the AFL with a density of less than one opponent (d = 0.80; 0.24–1.35). The percent of total disposals performed while running (dynamic) and stationary showed large between group effects, with the AFL generating greater dynamic disposals and fewer stationary relative to the semi-elite competition (Table 2). Taken together, the results indicate that disposals within the AFL are shaped by greater spatial and temporal constraints than those performed within the semi-elite competition.

## 4. Discussion

This study compared constraint characteristics sampled from match play in elite and semi-elite AF competitions. Results indicated competition differences, with elite AF match play generating a greater percent of total disposals performed under more pronounced temporal (possession time), and spatial (opponent density around ball carrier and disposals performed while running) constraints. Practitioners are encouraged to consider these results when designing representative training activities for players within these respective competitions, as the disposal behaviours observed within the semi-elite competition are likely to emerge from a less pronounced constraint boundary relative to those within the elite competition.

Typically, disposals performed within the 0–1 s temporal epoch are quick kicks from congestion (to clear space and gain territory) or handballs to a teammate to prevent the opponent from tackling [16]. These actions often emerge as a product of physical pressure imposed from an opponent, which is supported by our results given the greater percent of total disposals performed with three or more opponents surrounding the ball carrier in the AFL. This could be a product of both advanced strategy and physical prowess shown by players within the AFL, enabling more opponents to physically ‘rush’ the ball carrier in an attempt to win ball possession. Further, AFL coaches could be utilising strategies intended to pressure opponents when they are in possession of the ball in an attempt to ‘lock’ the ball in a certain area of the ground (attacking zone (inside forward 50m), for example) [17]. Given this, AFL players have to be attuned to disposal affordances shaped by considerable temporal and spatial constraints (either quick kicking or handballing to teammates in space). It is logical to suggest that training within this elite environment should represent these types of spatial and pressured constraints [18]. Practitioners within the AFL could therefore use these results as a basis for developing representative training activities, with players learning to attune to relevant disposal affordances under representative conditions [11]. Further, our data suggests that talent development within AF should appreciate the increased spatial and temporal constraints within the AFL relative to a semi-elite competition and attempt to account for this within training design when developing prospective AFL players.

The lower percent of total disposals performed with less than one opponent surrounding the ball carrier in the AFL further indicates a greater level of physical pressure imposed on the ball carrier relative to the semi-elite competition. Approximately 35% of disposals within the AFL were performed with less than one opponent within a 3 m radius of the ball carrier, contrasted to approximately 40% within the semi-elite competition. It would therefore be important for AFL players to learn how to exploit this physical pressure to create functional movement solutions. A potential solution to this movement problem would be what coaches colloquially refer to as ‘taking the tackle’, in which the player absorbs the physical pressure from their opponent, frees their hands and handballs (passes) the ball to a teammate in space. Conversely, the greater amount of physical pressure imposed from an opponent within the AFL could guide players to identify and run to space, which is a notion supported by our results. We found a greater percent of total disposals were performed within the AFL while running (dynamic) when contrasted to the semi-elite competition. This suggests that AFL players could be acknowledging the greater spatial constraints imposed on them during match play, using their physical capacities (individual constraints) to identify and exploit space [19].

We see our results implicating two areas specific to coaching in AF. Firstly, AFL coaches should appreciate the perceptual-motor re-calibration that is likely required by players transitioning from a semi-elite competition into the AFL and account for this within activities such players undertake when in an AFL environment. Specifically, the behaviours observed by players within a semi-elite competition are likely to be shaped by differing constraint characteristics, which likely affords them with more time and space, meaning observed movement outcomes should be viewed relative to this. Secondly, the data presented here could be used by coaches at both elite and semi-elite levels to develop training activities that represent the constraints experienced by players in competition. In doing so, players will likely become attuned to affordances representative of those experienced in competition [18]. In addition to coaching practices, our results also extend to sports performance analysts. Notably, we encourage analysts to align practices with theoretical constructs common to skill acquisition. This will assist with an appreciation of the environment or context in which a behaviour emerges, rather than sampling the isolated movement outcome [9]. As shown in this study, appreciating the constraints which shape movements provides practitioners with contextual relativity when basing judgements on movement outcomes observed during match play.

This study is not without limitations. Firstly, data was extracted from one club participating in both the elite and semi-elite competition, with the constraint characteristics potentially being impacted by the intended game style or instruction constraints used by coaches at that club. Future work is encouraged to sample the performance landscapes using a greater number of teams to gain a deeper insight into differing constraint boundaries, thereby reducing the potential impact of any instructional constraints. Secondly, the constraints chosen for this study were not intended to be exhaustive, rather ‘samples’ of those common to a game of AF. Future studies may look to add to the constraints listed here while concurrently investigating their characteristics specific to a disposal type (i.e., what are the constraints shaping a kick type or handball within AF and how do these differ relative to competition standard?). Further, the constraints described in this study are de-limited to those shaping “skill”. Future work may consider examining the constraints that shape the emergence of physical behaviours, such as the exertion of high-speed running efforts. This may continue our understanding of the constraints that shape a range of behaviours in AF, assisting with the continued development of more representative training activities.

## 5. Conclusions

Results of this study demonstrate that differences exist with regards to the constraint boundaries players have to manage within elite and semi-elite AF competitions. It appears that AFL players have to be attuned to disposal affordances while under greater temporal and spatial constraints relative to their semi-elite counterparts. Training and development should subsequently reflect this, both at the elite and semi-elite levels. It would be of interest for future work to examine the constraint differences between competition standards in other team sports to assist with the establishment of representative training activities beyond the confines of AF.

## 6. Practical Implications

- Disposals performed within the AFL are shaped by more pronounced temporal and spatial constraints relative to those within semi-elite competitions.
- Coaches at both AFL and semi-elite levels could use the data presented here to design training activities that afford representative constraint characteristics specific to elite and semi-elite levels.
- Sports performance analysts should align practices with theories common to skill acquisition, affording them with data of use for representative learning design.

## Figures and Tables

**Table 1:** The constraint matrix used within the current study.

Constraint Class	Constraint	Description	Sub-Category Label
Task	Possession time in general play	Time between a player obtaining and then disposing of the ball while in general play (i.e., not from a mark or free kick)	0–1 s 1–2 s 2–3 s >3 s
	Possession time from a stoppage in play (i.e., a “mark”)	Time between a player obtaining the ball from a stoppage in play (mark or free kick) and then disposing of it	0–1 s 1–2 s 2–3 s >3 s
	Disposal location	Location of each ball disposal (kick or handball) partitioned into four field locations	Defensive 50 Defensive Midfield Attacking Midfield Forward 50
Environmental	Target density	Number of opposition players within a 3 m radius of the intended disposal target	Uncontested (e.g., 1 vs. 0) Even (e.g., 1 vs. 1) Superior (e.g., 2 vs. 1) Inferior (e.g., 1 vs. 2)
	Ball carrier density	Number of opposition players within a 3 m radius of the ball carrier at ball disposal	<1 Opposition Player 1 Opposition Player 2 Opposition Players 3 Opposition Players >3 Opposition Players
Individual	Disposal movement	Locomotive state at point of ball disposal	Stationary (e.g., walking) Dynamic (e.g., running)

*Note:* “s” denotes seconds, “m” denotes metres.

**Table 2:** Descriptive statistics (mean ± standard deviation) of the constraint characteristics relative to competition.

Notation	Sub-Category Label	Elite	Semi-Elite	*d* (90% CI)
General play TIP	0–1 s	36.57 ± 4.85	31.21 ± 3.99	1.24 (0.64–1.80)
	1–2 s	25.52 ± 2.41	24.64 ± 2.27	0.42 (−0.11 to 0.95)
	2–3 s	6.83 ± 1.29	8.77 ± 2.09	1.01 (0.52–1.66)
	>3 s	4.32 ± 1.37	6.32 ± 2.14	1.10 (0.52–1.67)
Stoppage TIP	0–1 s	0.85 ± 0.46	1.13 ± 0.61	0.50 (−0.03–1.04)
	1–2 s	3.27 ± 1.16	3.07 ± 1.21	0.16 (−0.36–0.69)
	2–3 s	3.35 ± 1.04	3.61 ± 1.31	0.21 (−0.31–0.74)
	>3 s	19.15 ± 2.75	21.21 ± 3.21	0.69 (0.14–1.23)
Disposal location	Defensive 50	23.20 ± 4.47	25.48 ± 7.34	0.37 (−0.16–0.90)
	Defensive Mid	34.39 ± 3.75	35.53 ± 6.66	0.20 (−0.32–0.73)
	Attack Mid	29.15 ± 4.43	27.38 ± 5.91	0.33 (−0.19–0.87)
	Forward 50	13.23 ± 3.00	11.60 ± 3.94	0.46 (−0.07–1.00)
Target density	Uncontested	23.2 ± 7.53	23.6 ± 7.12	0.05 (−0.47–0.58)
	Even	47.07 ± 5.50	47.35 ± 6.40	0.04 (0.48–0.57)
	Inferior	23.73 ± 6.46	21.42 ± 4.89	0.41 (−0.12–0.94)
	Superior	5.95 ± 2.12	7.57 ± 3.28	0.58 (0.03 – 1.12)
Carrier density	<1 Opposition	35.58 ± 4.67	39.21 ± 4.47	0.80 (0.24–1.35)
	1 Opposition	29.03 ± 2.78	28.49 ± 2.91	0.19 (−0.34–0.72)
	2 Opposition	18.06 ± 2.11	18.13 ± 3.67	0.02 (−0.51–0.55)
	3 Opposition	9.61 ± 2.08	7.85 ± 2.21	0.82 (0.26–1.37)
	>3 Opposition	7.71 ± 3.09	6.30 ± 2.16	0.54 (0.01–1.08)
Disposal movement	Dynamic	89.39 ± 3.24	86.59 ± 3.24	0.89 (0.33–1.45)
	Stationary	10.60 ± 3.09	13.40 ± 3.09	0.89 (0.33–1.45)

*Note*: “TIP” denotes time in possession, “s” denotes seconds, “m” denotes metres and “Mid” denotes midfield.
